# Blue or far‐red light supplementation induced pre‐hardening in the leaves of the *Rht12* wheat dwarfing line: hormonal changes and freezing tolerance

**DOI:** 10.1111/ppl.70112

**Published:** 2025-03-03

**Authors:** Zsolt Gulyás, Mohamed Ahres, Tamás Pálmai, Kitti Kulman, Zahra Tahmasebi, Kalpita Singh, Kristóf Jobbágy, Danuše Tarkowská, Petre Dobrev, Radomíra Vanková, Péter Borbély, Andreas Börner, Gábor Galiba

**Affiliations:** ^1^ Agricultural Institute, Centre for Agricultural Research, HUN‐REN Martonvásár Hungary; ^2^ Doctoral School of Plant Sciences, MATE Hungarian University of Agricultural and Life Sciences Gödöllő Hungary; ^3^ Festetics Doctoral School, MATE Hungarian University of Agricultural and Life Sciences Keszthely Hungary; ^4^ Doctoral School of Biology and Institute of Biology, ELTE Eötvös Loránd University Budapest Hungary; ^5^ Laboratory of Growth Regulators, Faculty of Science, Palacký University Olomouc and Institute of Experimental Botany, the Czech Academy of Sciences Olomouc Czech Republic; ^6^ Laboratory of Hormonal Regulations in Plants, Institute of Experimental Botany of the Czech Academy of Sciences Prague Czech Republic; ^7^ Leibnitz Institute of Plant Genetics and Crop Plant Research Seeland Germany; ^8^ Department of Agronomy MATE Hungarian University of Agricultural and Life Sciences, GEORGIKON Campus Keszthely Hungary

## Abstract

Reduced height (*Rht*) genes have revolutionised wheat cultivation, but they can compromise freezing tolerance, and only a few alleles are in use. Thus, evaluating the role of other *Rht* alleles in stress responses is crucial. Far‐red supplementation of white light (W+FR) can induce pre‐hardening in cereals at 15°C. However, the relevant effect of blue light enrichment (W+B) is poorly described. This study investigates the influence of W+FR or W+B exposure in young winter wheat leaves of a tall (wild‐type, *rht12*) and a dwarf, gibberellin‐deficient (near‐isogenic line, *Rht12*) genotype in cv. Maris Huntsman background over 10 days at 15°C. The main objectives were to investigate the relationship between light quality, gibberellin homeostasis, and freezing tolerance. Key parameters such as frost injury, hormonal pools and the expression of relevant genes were examined. Results provided evidence about the involvement of *Rht* alleles in the basal freezing tolerance of wheat leaves from the side of gibberellin availability. It was revealed that W+FR and W+B treatments partially rescued the freezing‐sensitive phenotype of *Rht12* leaves, suggesting a potential compensatory mechanism. Analysis of gibberellic acid (GA) metabolism indicated differential responses to light treatments between the *Rht12* and wild‐type leaves, with implications for freezing tolerance. Moreover, alterations in hormone levels, including jasmonic acid (JA) and salicylic acid (SA), were observed, highlighting the complex interplay between light signalling and hormonal regulation in wheat. Overall, these findings suggest that manipulating light responses may offer a strategy to enhance freezing tolerance in gibberellin‐deficient dwarf wheat genotypes.

## 1 INTRODUCTION

In the 1960s, significant changes began in agriculture that resulted in increased wheat yields worldwide. During this period, known as the ‘Green Revolution’, Reduced height genes (*Rht*) were introduced into wheat breeding (Pingali, 2012). These genes induce a reduction in plant height, which contributes to lodging resistance when large doses of nitrogen fertilisers and irrigation are applied (Hedden, 2003). By the late 1990s, one of the two main semi‐dwarfing genes, *Rht1‐B1b* (*Rht1*) or *Rht1‐D1b* (*Rht2*), was present in 70% of wheat cultivars worldwide (Evans, 1998). The importance of *Rht* genes in wheat cultivation is shown by the emerging trend in the number of publications involving *Rht* alleles – including gene mapping and the investigation of agronomic traits or stress tolerance – in the past fifteen years.

*Rht* genes encode growth repressor DELLA proteins (DELLAs), which repress gibberellin (GA) signal transduction (Hussain & Peng, 2003). Mutant alleles of *Rht* have single nucleotide polymorphisms that result in insensitivity to GAs (Pearce et al., 2011). This insensitivity leads to the accumulation of DELLAs, which subsequently causes a reduction in stem height. However, it is worth noting that in the Mediterranean climate, where there often is less moisture in the upper part of the soil, the short coleoptile of *Rht* NILs containing DELLA mutation can be problematic, hindering seedling emergence and early vigour (Rebetzke et al., 2014). Thus, researchers began to explore alternative dwarfing alleles, which do not adversely affect coleoptile length (Gasperini et al., 2012; Ford et al., 2018; Buss et al., 2020). One of these *Rht* alleles, *Rht12* is a dominant dwarfing gene, which was described in a gamma‐ray‐induced Karcagi 522M7K hexaploid wheat mutant (Sutka & Kovács, 1987). GA‐responsive *Rht12* contributed to reduced height (40‐50%), delayed ear emergence time, and increased spikelet fertility without reduced coleoptile length (Worland et al., 1994). Nevertheless, *Rht12* showed increased grain yield, lodging resistance, and harvest index alongside a reduced grain weight (Rebetzke et al., 2012). Unlike *Rht1‐B1b* and *Rht1‐D1b*, the *Rht12* phenotype can be restored by exogenous bioactive GA, indicating a deficiency in GA biosynthesis (Börner et al., 1996; Chen et al., 2014). According to Korzun et al. (1997) and Sun et al. (2019), *Rht12* was mapped on the terminal end of chromosome 5AL, and the dwarfism was associated with the activation of the *TaGA2oxA14* gene (later *TaGA2oxidaseA13*). Buss et al. (2020) confirmed that the *TaGA2oxidaseA13* (*TaGA2oxA13*) is localized in the *Rht12* region on chromosome 5A. In addition, they suggested a height‐reducing mechanism through the increased expression level of *TaGA2oxA13* and lower GA_1_ content similar to *Rht18* and *Rht14*. However, while numerous studies suggest a remarkable potential for using *Rht12* in wheat breeding programs, this has not been realized yet (Worland et al., 1994; Chen et al., 2013). Additional stress sensitivity was indicated in gibberellin‐insensitive near‐isogenic lines (NILs) of wheat harbouring mutant *Rht* alleles *Rht‐B1b* and *Rht‐D1b* to heat (Cseh et al., 2024) or in the case of *Rht‐B1b* or *Rht‐B1c* to freezing (Szalai et al., 2022).

GAs are diterpenoid carboxylic acids that regulate several processes related to plant growth and development, such as seed germination, stem elongation, leaf expansion, transition the shoot apex from vegetative to generative phase to induce flowering, fruit development (Gupta & Chakrabarty 2013). GAs stimulate plant growth by overcoming the growth‐restraining effects of DELLAs. When the receptor protein GID1(GIBBERELLIN INSENSITIVE DWARF1) perceives bioactive GAs, a conformational change occurs in the N‐terminal region of GID1. It enhances the formation of the GA–GID1–DELLA complex (Ueguchi‐Tanaka et al., 2007; Murase et al., 2008). This enables the SCF (SKP1, CULLIN, F‐box) E3 ubiquitin ligase complex to recognize and polyubiquitinate DELLA, which leads to its degradation via the 26S proteasome pathway (Sasaki et al., 2003). DELLA targets are then released, promoting downstream GA‐mediated growth responses.

Numerous GAs have been identified in plants, but only four are biologically active – GA_1_, GA_3_, GA_4,_ and GA_7_ (MacMillan, 2001). In their complicated, multi‐level biosynthetic pathway, GAs are formed from *trans*‐geranylgeranyl diphosphate (GGPP), which is transformed to *ent*‐kaurene with the involvement of *ent*‐copalyl diphosphate synthase (CPS) and *ent*‐kaurene synthase (KS) (Yamaguchi, 2006). The cytochrome P450 monooxygenases such as *ent*‐kaurene oxidase (KO) and *ent*‐kaurenoic acid oxidase (KAO) catalyse the conversion of *ent*‐kauren to GA_12_ aldehyde. At this point in biosynthesis, the pathway splits into two: the 13‐hydroxylation pathway and the non‐13‐hydroxylation pathway. On the 13‐hydroxylation pathway, GA_1_ and GA_3_ form through GA_53_→ GA_44_→ GA_19_→ GA_20_, whereas another pathway results in GA_4_ and GA_7_ through GA_12_→GA_15_→ GA_24_→ GA_9_ with the participation of GA 20‐oxidase (GA20ox) and GA 3‐oxidases (GA3ox) operating in both pathways (Hedden, 2020). The GA deactivation reactions are catalysed by GA 2‐oxidases (GA2oxs) that belong, together with GA20ox and GA3ox, to the 2‐oxoglutarate‐dependent dioxygenases (2ODDs). These reactions result in, for example, GA_8_, GA_34_, and GA_29_ (Yamaguchi, 2008). According to Sun et al. (2019), the level of bioactive GAs (products of GA3ox) and inactive GA precursors (products of GA20ox) in the biosynthetic pathway were significantly lower in the *Rht12* dwarf line at both the jointing and the heading stages. They suggested that the *Rht12* dwarfing gene influences the GA biosynthetic and deactivation pathways by the overexpression of *TaGA2oxA13*, which hydroxylates the GA precursors at the early phase of the biosynthetic pathway. Buss et al. (2020) confirmed the involvement of *TaGA2oxA13* in the reduced height phenotype in *Rht12* lines and predicted that GA2oxA13 targets GA_12_, resulting in lower levels of this metabolite and higher levels of GA_110_ (2‐OH GA_12_).

Cold acclimation is a well‐known phenomenon in plants, which helps them adapt to cold by way of increased freezing tolerance and it is a requirement for the winter survival of winter habit cereals (Thomashow, 1999). One of the most significant transcription factor families in cold acclimation is the C‐REPEAT‐BINDING FACTOR (CBF) group, which regulates *COLD‐RESPONSIVE* (*COR*) genes, enhancing the freezing tolerance of both dicot and monocot plants (Jaglo‐Ottosen et al., 1998; Soltész et al., 2013). *CBF* gene induction has a temperature threshold, which is around 10 to 12°C in the case of winter wheat, rye or barley. To fully achieve cold acclimation in *Triticae*, the temperature must be below 10°C for 4‐6 weeks in short‐day photoperiod, so wheat could withstand approximately ‐9/‐18°C minimal temperatures (Caccialupi et al., 2023). Thus, not only the success of cold acclimation but also the genetic background specifically determines the winter survival of wheat (Vágújfalvi et al., 1999; Ganeva et al., 2013). However, shorter cold exposure (around 7 days), previously termed as pre‐hardening, also enhances the freezing tolerance of winter wheat or barley leaves to some extent (Novák et al., 2016), but it is insufficient for full plant tolerance if the soil freezes deep enough to damage the crowns (Vágújfalvi et al., 1999), because their acclimation does not occur under these conditions (Sutka, 1981). Besides the minimal tolerable temperatures, the other main difference between freezing tolerant and sensitive genotypes is that the tolerant plants are able to cold acclimate much quicker and start to acclimate at higher temperatures than the sensitive ones. This phenomenon is termed as ‘threshold induction temperature’ (Fowler et al., 1996). The basal freezing tolerance – freezing tolerance of plants acclimated to moderate temperature – of plants be they spring or winter genotypes is very similar – ranging around ‐4 to ‐6°C (Fowler, 2008; Kosová et al., 2012; Novák et al., 2016). Consequently, before the full development of cold acclimation, young cereals could be susceptible to the sudden occurrence of frosty temperatures in the autumn. Future climate models predict the increase of the frequency and strength of suddenly occurring extreme cold events. Additionally, warmer autumns and milder winters are also predicted, which, along with fluctuating temperatures during the winter, could greatly diminish cold acclimation (Hanslin & Mortensen, 2010; Vaitkevičiūtė et al., 2022; Caccialupi et al., 2023; Larran et al., 2023).

Freezing enhances the accumulation of reactive oxygen species (ROS), which could result in significant cellular damage in the case of improper cold acclimation (Heidarvand & Maali Amiri, 2010; Arora, 2018). When the ROS‐scavenging system becomes overwhelmed due to excessive ROS accumulation under severe stress, cellular components are damaged. For example, the polyunsaturated fatty‐acid lipid components are common targets for oxidation by ROS. Alché (2019) provided a very comprehensive review of the field of lipid peroxidation in plants. One of the most frequently investigated markers of lipid peroxidation is malondialdehyde (MDA), a reactive carbonyl species (RCS), which usually accumulates in good correlation with the severity of different types of stresses. The level of MDA is highly dependent on fatty acid desaturation and is itself cytotoxic.

In the autumn, the genetically determined basal freezing tolerance could be influenced by various environmental factors other than cold (e.g. day length, intensity‐ and quality of light) in young winter‐habit cereals (Ahres et al., 2020, 2025; Roeber et al., 2021). By the approach of autumn, the Red:Far‐red (R:FR) as well as the Red:Blue (R:B) ratios become lower in the temperate climate zone (Kotilainen et al., 2020). Thus, it is not surprising that light quality could influence plant metabolism significantly (Borbély et al., 2022). Consequently, winter cereals could use light quality changes to modulate their resilience to freezing.

Indeed, low R:FR ratio could induce pre‐hardening without cold, even at moderate temperatures. It was first shown by Franklin & Whitelam (2007), that the exposure of Arabidopsis plants to low R:FR ratio light at 16°C was able to induce the cold acclimation response to some extent. FR supplementation to white light (W+FR) also resulted in increased freezing tolerance in young wheat and barley leaves acclimated to 15°C after 10 days (Novák et al., 2016). W+FR induced pre‐hardening involved the CBF regulon in wheat and barley (Novák et al., 2016; Ahres et al., 2021, 2025; Prerostova et al., 2021). Interestingly, almost half of the cold‐induced genes in Arabidopsis are also light‐regulated (Franklin & Whitelam, 2007; Liu et al., 2019). W+FR could also enhance cold‐induced pre‐hardening on cereal leaves, but the effect of low temperature will become dominant beyond 7 days (Novák et al., 2016).

Interestingly, the capability of a cereal cultivar for the light‐induced pre‐hardening process at 15°C highly depends on its own frost resistance. For example, ‘Cheyenne’ – moderately frost tolerant winter wheat – median lethal temperature [LT50] = ‐16°C after 21 days of cold acclimation; (Fowler et al., 1996), while ‘Nure’ (winter barley) can survive ‐13°C (Francia et al., 2004). The light‐induced pre‐hardening measured on leaf segments was effective in winter wheat cv. Cheyenne ranged from ‐7 to ‐9°C, while this value for Nure was ‐7°C (Novák et al., 2016). The capability for W+FR induced pre‐hardening in wheat genotypes differing in winter hardiness was also shown recently (Ahres et al., 2025). In summary, this pre‐hardening effect results in a milder increase in freezing tolerance of winter wheat or barley leaves at 15°C, which means the decrease of the tolerable minimal temperature by approximately 1‐4°C depending on the genetic background (Novák et al., 2016). In Arabidopsis (Landsberg *erecta*), this hardening effect was similar to the effect of 1‐4 days of cold (4°C) exposure (Gilmour & Thomashow, 1991; Franklin & Whitelam, 2007).

The expression level of *HvCBF14*, which has a prominent role in frost tolerance, was enhanced by monochromatic blue light treatment as well at 15°C in cereals (Novák et al., 2017). Later, it was shown in barley and wheat that blue light (λ_max_=410 nm) supplementation of W+FR or monochromic blue light – λ_max_=410 nm; investigated only in wheat – could enhance the freezing tolerance of the leaves (Ahres et al., 2023, 2025). However, knowledge about the relevant signalling pathways in cereals is sparse (Crosatti et al., 1999; Novák et al., 2017; Ahres et al., 2023, 2025). Consequently, the pre‐hardening inducing effect of altered light quality on the leaves of winter cereals is beneficial in mid‐ or late autumn or early winter, when cold acclimation is undeveloped or in its initial phase, but sudden frost could be occurring. At this period, the soil usually does not freeze deeply enough to damage the crowns; therefore, the damage to the aboveground parts, like leaves, is more relevant.

Phytohormones play a pivotal role in regulating plant responses to changing environments, including cold acclimation (Kosová et al., 2012; Vanková et al., 2014) or light quality‐induced pre‐hardening. Besides, several studies revealed that light quality influences both GA metabolism and signalling (Yang et al., 2015; Matsuo et al., 2019; Xu et al., 2021), furthermore, GAs have a central role in the acclimation to changing environmental conditions such as cold or altered light conditions (Franklin, 2009; Song et al., 2019). Diminished bioactive GA accumulation at low temperatures, along with growth reduction, are considered as markers of cold acclimation (Hüner et al., 2016). In several plant species, overexpression of *CBFs* caused a significant decrease in the levels of bioactive GAs and growth, which was restored by exogenous GA, indicating a connection with the biosynthetic pathway (Hsieh et al., 2002; Shan et al., 2007; Soltész et al., 2013; Zhou et al., 2014). It was revealed that *GA2ox* expression was induced by the cold or *CBF* overexpression, which resulted in the deactivation of bioactive GAs (Achard et al., 2008; Soltész et al., 2013). Besides, overexpression of *CBF1* in Arabidopsis caused enhanced DELLA accumulation, suggesting the involvement of DELLAs in *CBF1*‐mediated cold stress response (Achard et al., 2008). Nevertheless, a modulatory effect of GA signalling on cold signalling can be assumed during short cold exposure of Arabidopsis leaves (Lantzouni et al., 2020).

Intriguingly, there are contradictions in the literature regarding the negative correlation between growth/GA accumulation and freezing tolerance. In Arabidopsis, the overexpression of the DWARF AND DELAYED FLOWERING 1 (DDF1) transcription factor, a member of the CBF/DREB superfamily, resulted in a dwarf, GA‐sensitive mutant with enhanced stress tolerance, including freezing. While exogenous GA_3_ restored the dwarf and heat‐tolerant phenotype of the mutant, it only partially restored the freezing or drought tolerance and did not express a significant effect on the freezing tolerance of the wild‐type plants (Kang et al., 2011). Contrastingly, brassinosteroid (BS)‐deficient (BW084) and BS‐insensitive (BW312) dwarf, spring barley NILs exhibited reduced freezing tolerance after cold acclimation compared to the wild‐type background cv. Bowman (Sadura et al., 2019). Similarly, GA‐insensitive, semi‐dwarf (*Rht‐B1b*) and dwarf (*Rht‐B1c*) NILs in the facultative wheat cv. April Bearded background showed diminished freezing tolerance after cold acclimation, with the dwarf genotype being the most susceptible (Szalai et al., 2022). These findings suggest that dwarf phenotypes and/or impaired GA signalling do not necessarily enhance freezing tolerance. Additionally, plants illuminated with W+FR light do grow (Beall et al., 1996; Novák et al., 2016; Romanowski et al., 2021) with the involvement of GA accumulation (Beall et al., 1996; Wang et al., 2024), which also supports this hypothesis.

Contrasting patterns of GA_1_ and GA_3_ accumulation were observed in the aerial parts and crowns of spring barley cultivars Bowman (more freezing tolerant) and Delisa (less tolerant) after 3 weeks of cold acclimation (Sadura et al., 2019; Pociecha et al., 2020). In the aerial parts, GA_1_ decreased in both cultivars, while GA_3_ only decreased in Delisa. In the crowns, Bowman showed increased GA_3_ and diminished GA_1_ accumulation, while Delisa had increased GA_1_. Kosová et al. (2012) described the time course of hormonal remodelling and bioactive GA accumulation during the different phases of cold acclimation in the fully developed leaves of winter and spring wheat. They found enhanced bioactive GA levels during the pre‐hardening phase (7 days), followed by a decrease after 3 weeks at low temperatures. Distinct accumulation patterns of bioactive GAs were also observed between winter and spring genotypes. These findings also highlight the importance of a deeper understanding of the role of GAs and *Rht* alleles in different phases of cold acclimation and predict their importance in light quality‐induced pre‐hardening.

To date, we have no information about how the *Rht* allele‐related GA‐deficiency would affect the light quality‐induced pre‐hardening in wheat. Thus, the aim of this study was to investigate the effects of blue (W+B) or far‐red (W+FR) supplementation of white light (W) on winter wheat and the role of GA accumulation in light quality‐induced pre‐hardening. For this, a tall cultivar Maris Huntsman (MH) – undisturbed GA biosynthesis; wild‐type *rht12* allele – and a GA‐sensitive, dwarf NIL in MH background – diminished GA accumulation; mutant *Rht12* allele – were used to evaluate the basal freezing tolerance of the NIL and whether changing light quality rescues the dwarf phenotype or affects its response to freezing. The influence of specific light treatments – provided by artificial LED light sources for 10 days at 15°C – on leaf frost injury, MDA accumulation, GA metabolism, and the expression levels of GA biosynthesis‐ or cold acclimation‐related genes as well as other hormonal changes (abscisic acid – ABA, jasmonic acid – JA, salicylic acid – SA, auxin indole‐3‐acetic acid – IAA, cytokinins – CK) were monitored.

## 2 MATERIALS AND METHODS

### 2.1 Plant materials and growth conditions

Tall, wild‐type winter wheat *Triticum aestivum* ssp. *aestivum* cv. Maris Huntsman (containing the recessive, wild‐type *rht12* allele) and the NIL containing the dominant *Rht12* mutant allele were used in our experiments. Maris Huntsman (MH) is a winter wheat genotype that originated in the UK (1960s). This old wheat variety has moderate frost tolerance, which is suitable for use in the United Kingdom's climate, and its wild‐type genotype does not contain mutations in green revolution genes (Prášilová & Prášil, 2001). The *Rht12* source material, Karcagi 522M7K is a short, winter habit wheat reselected for stability by Konzak et al. (1984) from the Karcagi 522 mutant population induced by γ‐radiation of dry seeds (Viglási, 1968). The *Rht12* NIL was developed by backcrossing Karcagi 522M7K into MH four times (Worland et al., 1994). *Rht12* and *rht12* (MH) seeds were acquired from the Gene bank of the Leibniz Institute of Plant Genetics and Crop Plant Research (IPK) in Gatersleben, Germany. After germination, seedlings were grown in wooden boxes for two weeks in a PGV‐36 growth chamber (Conviron PGV36; Controlled Environments Ltd.) under 12:12 h photoperiod (250 μmol m^−2^ s^−1^, constant 15°C, 70/75% relative humidity) equipped with a modular LED light ceiling. The illumination was provided by a continuous wide‐spectrum LED (Philips Lumileds, LXZ25790‐y), which was considered as our control W light. The plants received irrigation using 50% Hoagland medium three times per week (Ahres et al., 2023).

### 2.2 Conditions of light and temperature treatments

**FIGURE 1:**
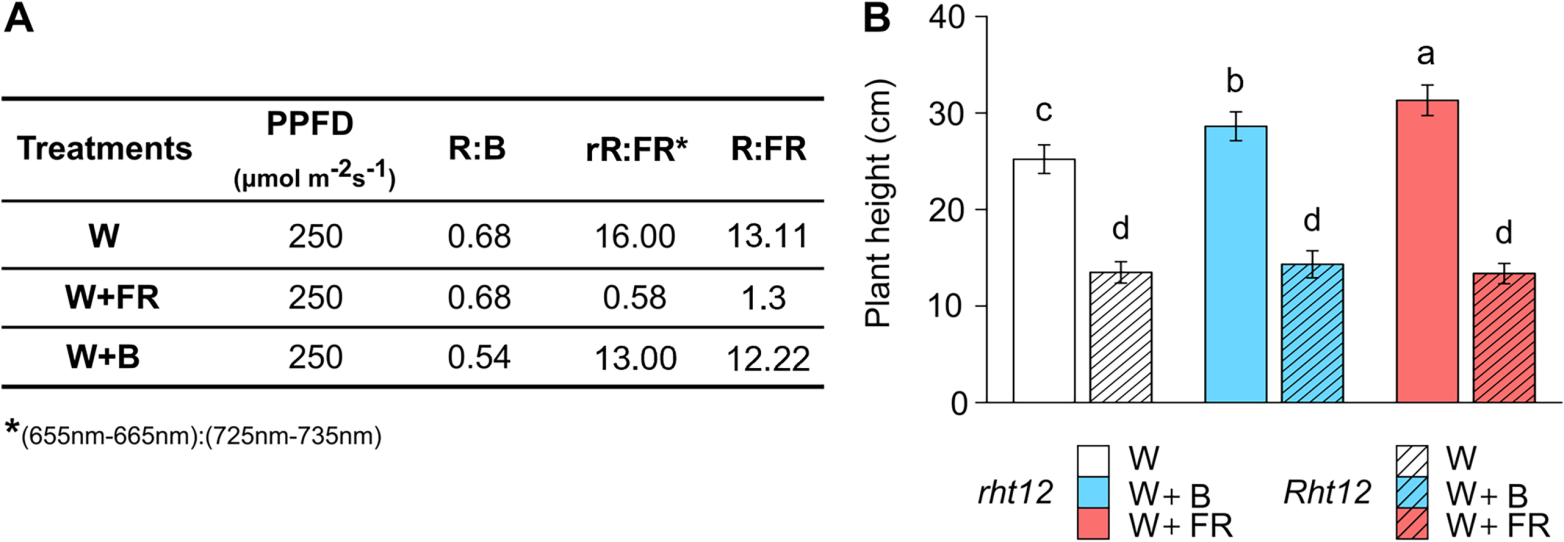
Details of the light conditions (A) and the effect of modified spectral conditions on the height of the wheat plants (B) after 10 days at 15°C. W: white light control (white colour), W+B: blue light enrichment (blue colour), W+FR: far‐red light enrichment (red colour). Solid bars: *rht12*; stripped bars: *Rht12*. Values marked with different letters are significantly different from each other at the level of *p*≤0.05 (n=15).

Following the two‐week developmental phase, the plants were in the Z12 (Zadok's scale) growth stage. The plants were then separated into three zones. In the first zone with White (W) light at 250 μmol m^−2^ s^−1^, intensity was unchanged. In the second zone, W light was supplemented with far‐red illumination (W+FR) using a narrow‐band 750 nm LED (Edison Edixeon, 2ER101FX00000001), decreasing the R:FR ratio (600‐700:700‐800 nm) to 1.3. The rR:FR ratio (655‐665:725‐735 nm=0.58) was calculated based on Smith's method (Smith, 1982). In the third zone, W intensity was reduced, and W was supplemented with blue light (W+B) using a very narrow (λ_max_=410 nm) monochromatic LED (Philips Lumileds, LXZ1‐PR01) to maintain the same light intensity but with a lowered R:B ratio (Figure 1A). The duration of the light treatments was 10 days at 15°C. The spectral composition of applied light treatments is provided in the supplementary material (Figure S1).

### 2.3 Leaf freezing tests and electrolyte leakage measurements (Degree of frost injury)

To determine the freezing tolerance of leaves, we performed the protocol outlined in Webb et al. (1994) modified by the guidance of Prášil and Zámečník (1998). This methodology uses excised leaf segments and provides a good correlation with the cold acclimation level of the leaves (Webb et al. 1994), and could provide a good estimate of the survival of intact leaf tissues under conditions when the cold acclimation of cereal crowns are not relevant (Bannister, 2007; Franklin & Whitelam, 2007; Min et al., 2020).

Freezing protocols were implemented using a GP200‐R4 liquid freezing system (Grant Instruments), as detailed in our prior publication (Ahres et al., 2021). Briefly, approximately two‐mm‐long leaf segments of equal size were excised from one leaf (completely developed true leaves), each of four plants per genotype in every light treatment and placed in 14‐mL, empty Falcon tubes (Thermo Fisher Scientific Inc.). To accurately determine the degree of frost injury, a set of these samples was collected for each temperature from these same leaves: 1×4 segments for the unfrozen sample, 2×1×4 segments for the frost‐treated samples (‐4 and ‐6°C) and 1×4 segments for the frost‐killed ones. From this set of samples, seven biological replicates were applied, which means that a total of 4×7=28 plants/genotype/light treatment was used during the freezing tests. After the initial temperature decrease and ice nucleation‐inducing steps (see Ahres et al., 2021), the samples were incubated at ‐2°C for 18 h and the final selected temperatures for freezing were ‐4 and ‐6°C (for 1 h), which represents a tolerable and severe freezing stress to the wild‐type leaves, respectively (based on preliminary results).

Using electrical conductivity to determine the condition of the frost‐treated samples is common in freezing stress experiments, and with a careful application, the results are in good agreement with cell membrane damage and winter hardiness or survival data (Webb et al., 1994; Prášil & Zámečník, 1998; Nesbitt et al., 2002; Sun et al., 2017; Wang et al., 2017). Subsequently, to the freezing exposure, 8 mL of ultra‐pure water was added to the samples, which were then rinsed for 2 h at room temperature (in the dark). After the incubation, the electrical conductivity of the incubation fluid was measured using a conductometer (Mikro KKT). The degree of frost injury of the frost‐treated leaves was calculated using the electrolyte leakage data of the frost‐treated, unfrozen and frost‐killed (several freeze‐thaw cycles in liquid nitrogen) samples according to the formula proposed by Prášil and Zámečník (1998). This formula reduces the level of measurement errors originating from the methodology (e.g. unequal electrolyte content of the samples due to treatments or genotype). The electrical conductivity protocol was implemented on the unfrozen samples immediately after the sampling. For data analysis, Multi‐Sample Conductometer version 1.0 (Intron Software, Biological Research Centre, Szeged, Hungary; Copyright© L. Menczel, 2002) was used. Values above 60% indicate severe damage in the tissues (Nesbitt et al., 2002; Sun et al., 2017; Wang et al., 2017).

### 2.4 Measurement of lipid peroxidation

The MDA level in shoot samples was measured to estimate lipid peroxidation, following the method described by Asghar et al. (2023). Leaf segments were collected from four plants in three biological repeats and placed into falcon tubes. The frost treatments were implemented as described above (GP200‐R4 liquid freezing system, Ahres et al. 2021). After frost exposure, the samples were immediately processed. Lipid peroxide concentrations, expressed as MDA levels, were calculated using an extinction coefficient of 155 mM^‐1^cm^‐1^ (De Paula et al., 1996).

### 2.5 Hormone analysis

Phytohormones were analysed according to Prerostova et al. (2021). Frozen leaf samples (total n=5; approx. 10 mg FW) were homogenised with zirconia beads in a FastPrep‐24 5G homogeniser (MP Biomedicals) for 40 s at 6 m s^‐1^. Phytohormones were extracted twice with 100 μL 1 M formic acid. Isotope‐labelled standards (10 pmol/sample) were added to samples: ^13^C_6_‐IAA, ^2^H_4_‐OxIAA, ^2^H_4_‐OxIAA‐GE (Cambridge Isotope Laboratories); ^2^H_4_‐SA, ^2^H_2_‐GA19 (Sigma‐Aldrich); ^2^H_3_‐PA, ^2^H_3_‐DPA (NRC‐PBI); ^2^H_6_‐ABA, ^2^H_5_‐JA, ^2^H_5_‐tZ, ^2^H_5_‐tZR, ^2^H_5_‐tZRMP, ^2^H_5_‐tZ7G, ^2^H_5_‐tZ9G, ^2^H_5_‐tZOG, ^2^H_5_‐tZROG, ^15^N_4_‐cZ, ^2^H_3_‐DZ, ^2^H_3_‐DZR, ^2^H_3_‐DZ9G, ^2^H_3_‐DZRMP, ^2^H_7_‐DZOG, ^2^H_6_‐iP, ^2^H_6_‐iPR, ^2^H_6_‐iP7G, ^2^H_6_‐iP9G, ^2^H_6_‐iPRMP (Olchemim). The extracts were centrifuged at 4°C and 30,000 *g*. The supernatants were applied to the SPE Oasis HLB 96‐well column plate (10 mg/well; Waters) activated with 100 μL 50% acetonitrile and 100 μL 1 M formic acid. Elution was performed with 100 μL 50% acetonitrile using a Pressure+ 96 manifold (Biotage). The sediments were re‐extracted with 100 μL 1 M formic acid, the supernatants were centrifuged and applied again to the column plate.

Phytohormones were separated on a Kinetex EVO C18 column (2.6 μm, 150 × 2.1 mm, Phenomenex). The mobile phase consisted of A – 5 mM ammonium acetate and 2 μM medronic acid in water, and B – 95:5 acetonitrile: water (v/v). The following gradient program was applied: 5% B in 0 min, 7% B in 0.1 min to 5 min, 10 to 35% in 5.1 min to 12 min, 100% B in 13 to 14 min, and 5% B in 14.1 min. Hormone analysis was performed using an LC/MS system consisting of a UHPLC 1290 Infinity II (Agilent) coupled to a 6495 Triple Quadrupole Mass Spectrometer (Agilent). MS analysis was performed in MRM mode using the isotope dilution method. Data processing was performed with Mass Hunter Software B.08 (Agilent).

### 2.6 Analysis of GAs

Sample preparation and GA analysis were performed according to the method described by Urbanová et al. (2013) with some modifications. Briefly, tissue samples of about 30 mg FW were ground to a fine consistency using 2.7‐mm zirconium oxide beads (Retsch GmbH & Co. KG) and an MM 400 vibration mill at a frequency of 27 Hz for 3 min (Retsch GmbH & Co. KG) with 1 mL of ice‐cold 80% acetonitrile containing 5% formic acid as extraction solution. The samples were then extracted overnight at 4°C using a benchtop laboratory rotator Stuart SB3 (Bibby Scientific Ltd.) after adding internal GA standards: [^2^H_2_]GA_1_, [^2^H_2_]GA_4_, [^2^H_2_]GA_6_, [^2^H_2_]GA_9_, [^2^H_2_]GA_19_, [^2^H_2_]GA_20_, [^2^H_2_]GA_29_ and [^2^H_2_]GA_44_ (OlChemIm, Czech Republic). The homogenates were centrifuged at 36,670 *g* and 4°C for 10 min, and corresponding supernatants were further purified using mixed‐mode SPE cartridges (Waters) and analysed by ultra‐high performance liquid chromatography‐tandem mass spectrometry (UHPLC‐MS/MS; Micromass). GAs were detected using multiple‐reaction monitoring mode of the transition of the ion [M–H]^−^ to the appropriate product. Masslynx 4.2 software (Waters) was used to analyse the data, and the standard isotope dilution method (Rittenberg & Foster, 1940) was used to quantify the GA levels.

### 2.7 Gene expression determination (RTq‐PCR)

For gene expression measurements, 50 mg leaf samples were collected from all treatments in three replicates. Total RNA was extracted using the Direct‐zolTM RNA MiniPrep kit (Zymo Research Corp.), and its concentration was quantified using a NanoDrop 2000 Spectrophotometer (Thermo Fisher Scientific Inc.). The cDNA samples were prepared by following the manufacturer's protocol, using Moloney Murine Leukemia Virus (M‐MLV) Reverse Transcriptase and oligo (dT)_18_ primer (Promega Corporation). For the analysis of gene expression patterns CFX96 TouchTM real‐time PCR Detection System (Bio‐Rad Hungary Ltd.), and qPCRBIO SyGreen Blue Mix (PCR Biosystems Ltd.) were used. Regarding the qPCR primers, a combination of custom‐designed primers and previously published primers were used (Table S1) (Zhang et al., 2007; Dhillon et al., 2010; Guo et al., 2019; Wang et al., 2023a). Relative expression levels were determined using the ΔΔCt method (Livak & Schmittgen, 2001), with *Ta54948* (Accession: XM_044601429) and *Ta4045* (Accession: XM_044487010) used as the reference genes (Paolacci et al., 2009).

### 2.8 Statistical analysis

Statistical analysis of the data was done using OriginPro software (OriginLab Corp.). The homogeneity of variances was checked using Levene's test, and normality was tested by the Kolmogorov–Smirnov probe. Analysis of variance (ANOVA) and post hoc comparisons of means were conducted, applying Tukey's honestly significant difference test. Fisher's least significant difference (LSD) test was used in the statistical analysis of gene expression data. Correlation analysis was performed using Pearson's correlation coefficient in Origin (Pro) Software (version 2021, OriginLab Corp.), and the network was created with yED Graph Editor (yWorks GmbH).

## 3 RESULTS

### 3.1 The effect of light supplementation on the height of Rht12 plants

In our experiments, the height of *Rht12* plants was reduced by ~48% under control conditions. Out of W+FR and W+B supplementation (Figure 1B), W+FR influenced the height in the *rht12* wild‐type, increasing it by ~25%, and W+B by ~10%. Interestingly, these modifications in the light spectra were ineffective in the case of the *Rht12* dwarfing line (Figure 1B).

### 3.2 The effect of light spectrum modifications on freezing tolerance

**FIGURE 2:**
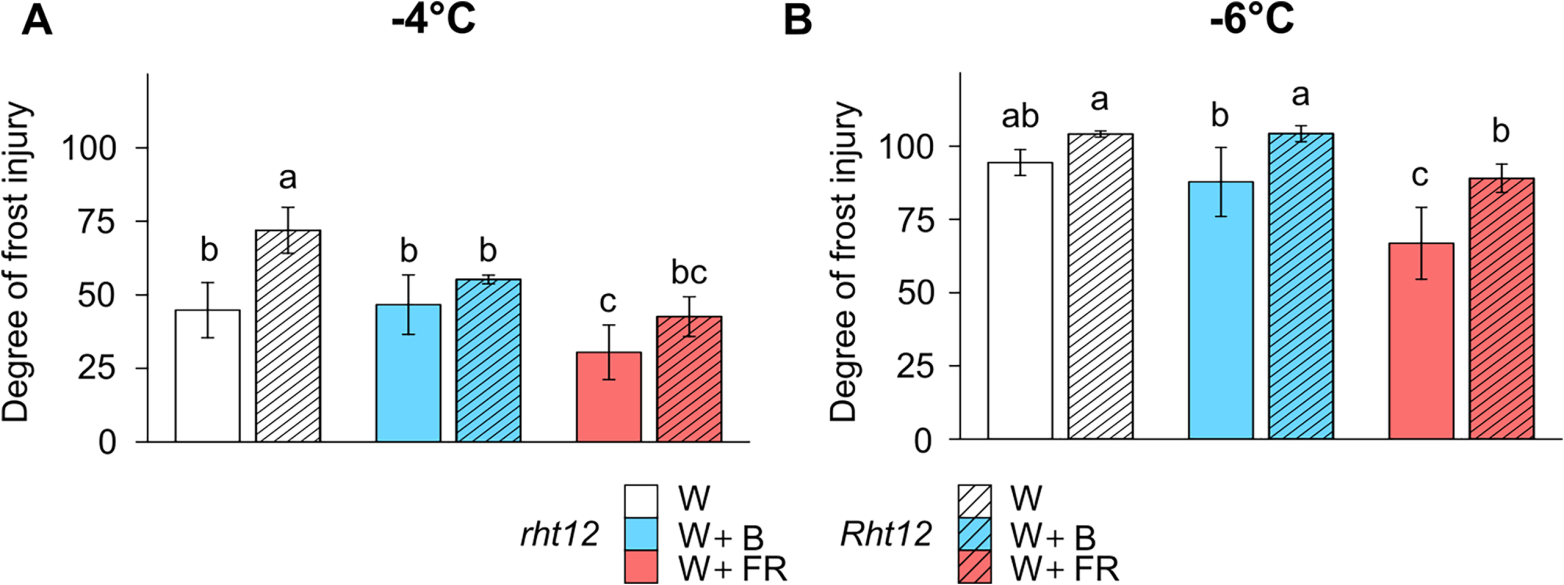
The impact of modified spectral conditions on the degree of frost injury at ‐4°C (A) and at ‐6°C (B) in the tall (*rht12*) and dwarf (*Rht12*) wheat leaf segments. The samples were collected after 10 days of light supplementation treatments at 15°C and subjected to freezing tests prior to relative conductivity measurements. W: white light (control, white colour), W+B: blue light enrichment (blue colour), W+FR: far‐red light enrichment (red colour). Solid bars: *rht12*; stripped bars: *Rht12*. Values marked with different letters are significantly different from each other at the level of *p*≤0.05 (n=7).

To investigate the effect of modulated light spectra on the freezing tolerance of *Rht12* and *rht12* leaves, we measured the leakage of electrolytes from the cells after exposing excised wheat leaf segments to frost. Then, we estimated the degree of frost injury (Figure 2), which indicates severe tissue damage at higher levels, as described in section 2.3. After W illumination, at ‐4°C, the wild‐type *rht12* leaf segments suffered ~50% frost injury (Figure 2A). In contrast, *Rht12* leaves were more sensitive, reaching about 75%. W+B light restored the frost tolerance of the *Rht12* leaves to the *rht12* wild‐type level, whereas W+FR treatment improved the freezing tolerance in the leaves of both lines. The significant positive effect of FR enrichment on freezing tolerance was indicated by the observation that electrolyte leakage from *Rht12* tissues decreased to the level of W light‐treated *rht12* leaves. A minimal temperature of ‐6°C was lethal for both genotypes after W and W+B light treatment. However, following FR enrichment, the degree of frost injury decreased in *rht12* leaves to around 60% compared to W treated wild‐type leaves but was unable to restore the freezing tolerance in *Rht12* significantly.

### 3.3 The effect of light spectrum modification on lipid peroxidation

**FIGURE 3:**
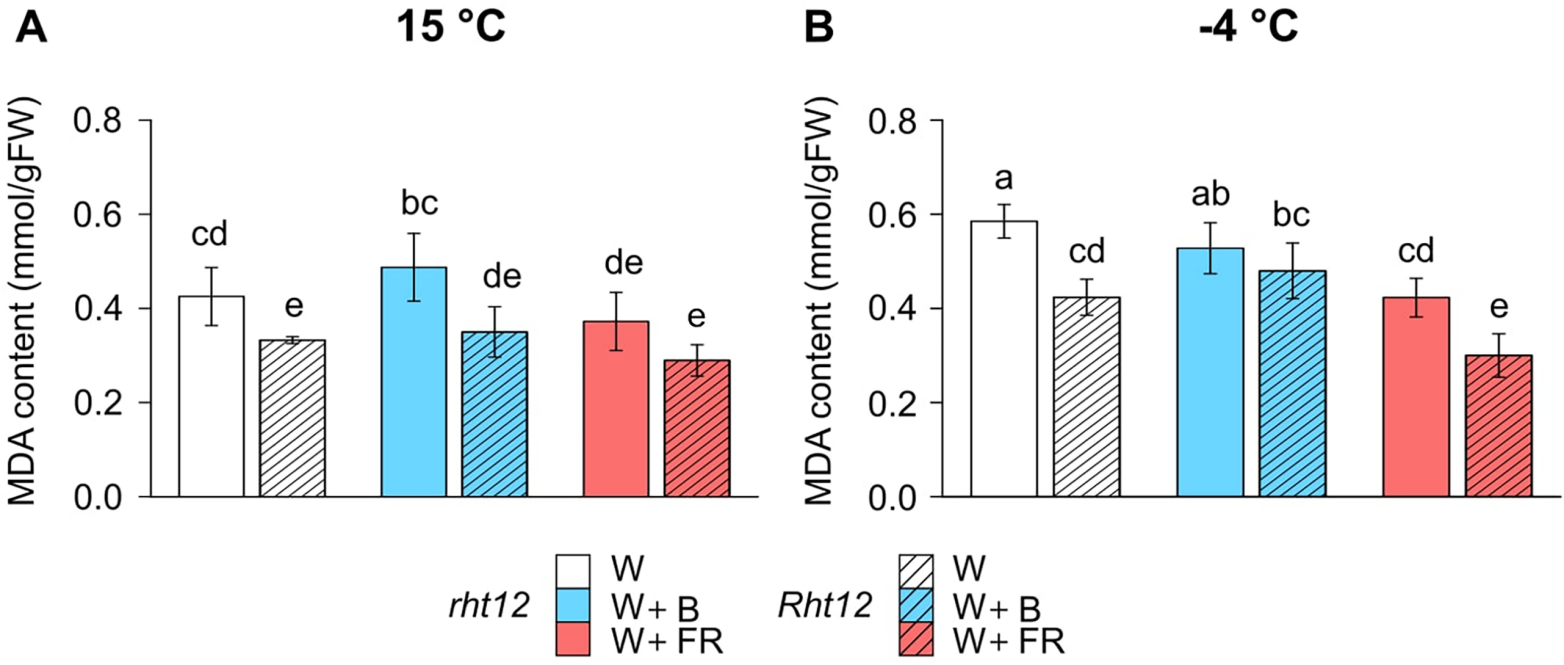
The effect of modified spectral conditions on lipid peroxidation at 15°C growing‐ (A) and ‐4°C minimal freezing temperatures (B) in the tall (*rht12*) and dwarf (*Rht12*) wheat leaf segments. The samples were collected to measure MDA content after 10 days of light supplementation treatments at 15°C and after the freezing test at ‐4°C. W: white light (control, white colour), W+B: blue light enrichment (blue colour), W+FR: far‐red light enrichment (red colour). Solid bars: *rht12*; stripped bars: *Rht12*. Values marked with different letters are significantly different from each other at the level of *p*≤0.05 (n=3). The statistical analysis of the data sets from parts A and B of the figure is common.

To characterize the extent of oxidative damage on lipids by ROS during freezing, MDA content was measured in leaf segments (Figure 3). Interestingly, MDA accumulation was significantly lower in *Rht12* leaves at 15°C compared to wild type under control conditions (Figure 3A). Light treatments did not alter MDA concentrations in *rht12* or in *Rht12* compared to their respective W‐treated samples. However, after W+FR treatment, the significant difference between *rht12* and *Rht12* leaves, which was observed under W illumination, vanished at 15°C. Similarly, MDA content was significantly lower in the leaf segments of the W treated dwarfing line compared to the wild type after ‐4°C freezing exposure (Figure 3B). Nevertheless, the mean increases in MDA levels in the *rht12* and *Rht12* lines compared to their own unfrozen MDA concentrations in W light treated leaves showed little difference, ~37.5 and ~27.5%, respectively. W+FR treatment significantly reduced the MDA content after freezing at ‐4°C in both genotypes compared to control illumination, whereas W+B did not affect the MDA accumulation of the samples. Considering the freezing injury results, the ‐6°C minimal temperature was too severe for the leaves, and MDA levels showed inconsistent changes with frost injury (Figures 2B & S2).

### 3.4 The effect of light supplementation on the GA biosynthetic pathway

**FIGURE 4:**
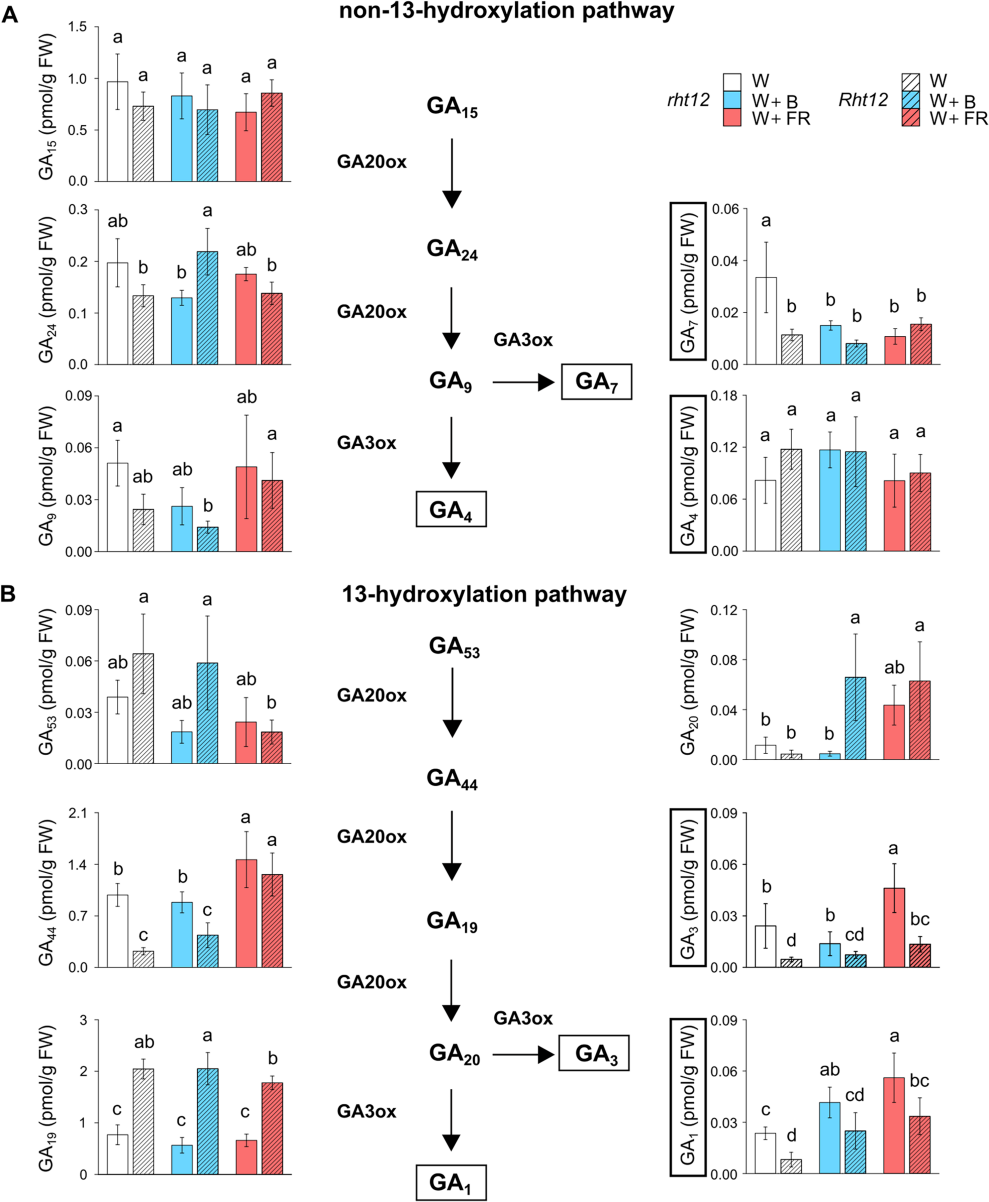
The effect of modified spectral conditions on GA contents in the non‐13‐hydroxylation pathway (A) and the 13‐hydroxylation pathway (B) in the tall (*rht12*) and dwarf (*Rht12*) wheat leaves after 10 days at 15°C. W: white light (control, white colour), W+B: blue light enrichment (blue colour), W+FR: far‐red light enrichment (red colour). Solid bars: *rht12*; stripped bars: *Rht12*. The bioactive GAs, namely GA_1_, GA_3_, GA_4_ and GA_7_ are highlighted with text boxes. Values marked with different letters are significantly different from each other at the level of *p*≤0.05 (n=5).

The *Rht12* mutation affects GA biosynthetic and deactivation pathways. On this basis, a quantitative analysis of GAs was performed in our experimental system. The bioactive GAs produced by the non‐13‐hydroxylation pathway, GA_7_ and GA_4_ responded differently to the modification of the light spectrum (Figure 4A). Under W light, GA_7_ content was significantly lower (by ~68%) in the *Rht12* line compared to the *rht12* wild type. While neither the *Rht12* allele nor the light treatments affect GA_4_ content significantly, both B and FR supplementation reduced GA_7_ content in *rht12* to the level of the dwarfing line. The levels of GA_15_ or GA_9_, bioactive GA precursors in this pathway, were not affected by the treatments in the leaves of either the tall or the dwarf genotype. However, under W+B light, GA_24_ content increased in *Rht12* leaves compared to the wild type.

In the 13‐hydroxylation pathway, the concentrations of bioactive GA_1_ and GA_3_ showed almost similar tendencies following the treatments (Figure 4B). In the leaves of the *Rht12* NIL, their amount was lower by ~70% than in the *rht12* leaves under W. The GA_1_ level was increased by B or FR light supplementations in *rht12* leaves compared to control illumination, whereas in the *Rht12* line, W+FR treatment had a significantly positive effect on its content compared to the leaves of the W‐treated NIL, which means the restoration of GA_1_ accumulation to the level of wild type under W light. In the case of GA_3_, W+B exposure did not have any effect, whereas W+FR treatment increased its content in both lines compared to their own genotype under control illumination. However, GA_3_ content was still significantly lower in *Rht12* leaves after W+FR treatment compared to the level of wild‐type leaves treated with W. Among the precursors of GA_1_ and GA_3_, GA_53_ was not affected by the mutation or light supplementation treatments. Nevertheless, GA_44_ content was lower in the *Rht12* line than in the wild type under W and W+B, but W+FR significantly increased GA_44_ accumulation in both lines above the level of *rht12* observed under control illumination.

### 3.5 The effect of light supplementation on the deactivated GA forms

**FIGURE 5:**
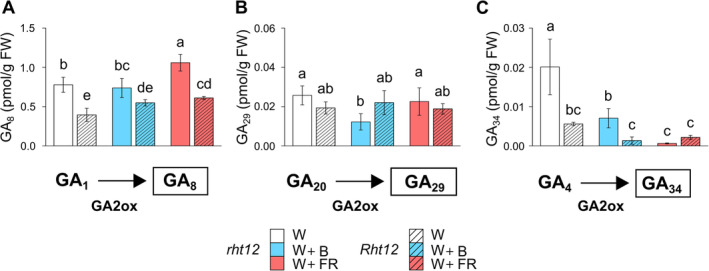
The effect of modified spectral conditions on GA deactivation pathways, namely GA_8_ (A), GA_29_ (B), and GA_34_ (C) in the tall (*rht12*) and dwarf (*Rht12*) wheat leaves after 10 days at 15°C. W: white light (control, white colour), W+B: blue light enrichment (blue colour), W+FR: far‐red light enrichment (red colour). Solid bars: *rht12*; stripped bars: *Rht12*. Values marked with different letters are significantly different from each other at the level of *p*≤0.05 (n=5).

The mechanisms of GA inactivation play a crucial role in enabling plants to adapt to the continuously changing environment. During this process, bioactive GA content is reduced, leading to the elevated accumulation of deactivated GAs. In our experiments, we measured the content of three deactivated forms, namely GA_8_, GA_29_, and GA_34_ (Figure 5). The level of GA_8_ and GA_34_ was significantly lower in *Rht12* leaves than in the wild type under every light condition. The levels of GA_29_ and GA_34_ were significantly reduced in the *rht12* leaves by B supplementation compared to the control conditions. Under W+FR light, GA_8_ content significantly increased in both *Rht12* and *rht12* leaves compared to their own genotype under control conditions. Nevertheless, the accumulation level of GA_8_ in *Rht12* leaves still remained below *rht12* treated with W. Similarly to W+B exposure, GA_34_ content was decreased by W+FR treatment in *rht12* to the level of the dwarfing line.

### 3.6 Impact of the light supplementation on the accumulation of other phytohormones

**FIGURE 6:**
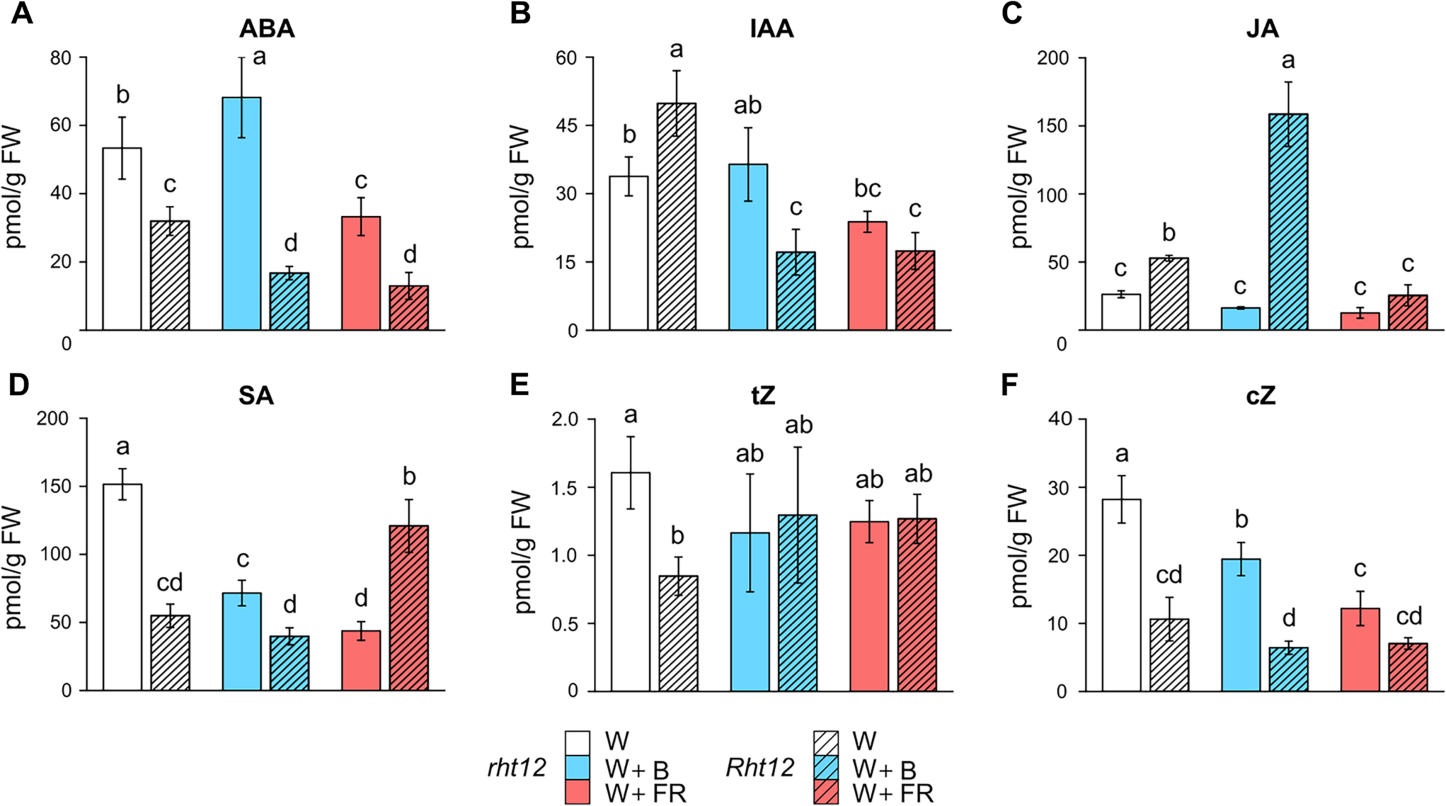
The impact of modified spectral conditions on hormone concentrations: abscisic acid (ABA, A); auxin (IAA, B); jasmonic acid (JA, C); salicylic acid (SA, D); *trans*‐zeatin (tZ, E); *cis*‐zeatin (cZ, F) in the tall (*rht12*) and dwarf (*Rht12*) wheat leaves after 10 days at 15°C. W: white light (control, white colour), W+B: blue light enrichment (blue colour), W+FR: far‐red light enrichment (red colour). Solid bars: *rht12*; stripped bars: *Rht12*. Values marked with different letters are significantly different from each other at the level of *p*≤0.05 (n=5).

Since there is comprehensive crosstalk among phytohormones in the regulation of cold acclimation, the amount of six hormones (Figure 6) and their metabolites (Table S2) was measured after the treatments. In *Rht12* leaves, the levels of abscisic acid (ABA), salicylic acid (SA), *trans*‐zeatin (*t*Z), and *cis*‐zeatin (*c*Z) were lower than those in wild‐type plants under control conditions (Figure 6). Contrastingly, the levels of bioactive auxin (IAA) and jasmonic acid (JA) showed an opposite tendency. After the W+B treatment, ABA content in *rht12* leaves increased significantly, whereas a remarkable decrease was observed in the dwarf line compared to their own genotype under control illumination. Interestingly, the W+FR treatment had a strong negative effect on ABA accumulation in both genotypes compared to W light (Figure 6A). In contrast to the W control, FR and B enrichment reduced auxin concentration in the *Rht12* leaves below the level observed in *rht12* under control conditions (Figure 6B). While B and FR enrichments did not affect JA accumulation in wild‐type leaves, a threefold increase in JA concentration was observed in the dwarf line following W+B treatment (Figure 6C). Both light supplementations decreased SA content significantly in *rht12* leaves, while W+FR treatment greatly increased the SA accumulation of *Rht12* leaves compared to the level observed under W exposure (Figure 6D). While the treatments had no impact on the *t*Z content of the lines, *c*Z levels were lower after the W+B and W+FR treatments in the case of *rht12* (Figure 6ef).

### 3.7 The expression patterns of gibberellin metabolism and cold acclimation‐related genes

**FIGURE 7:**
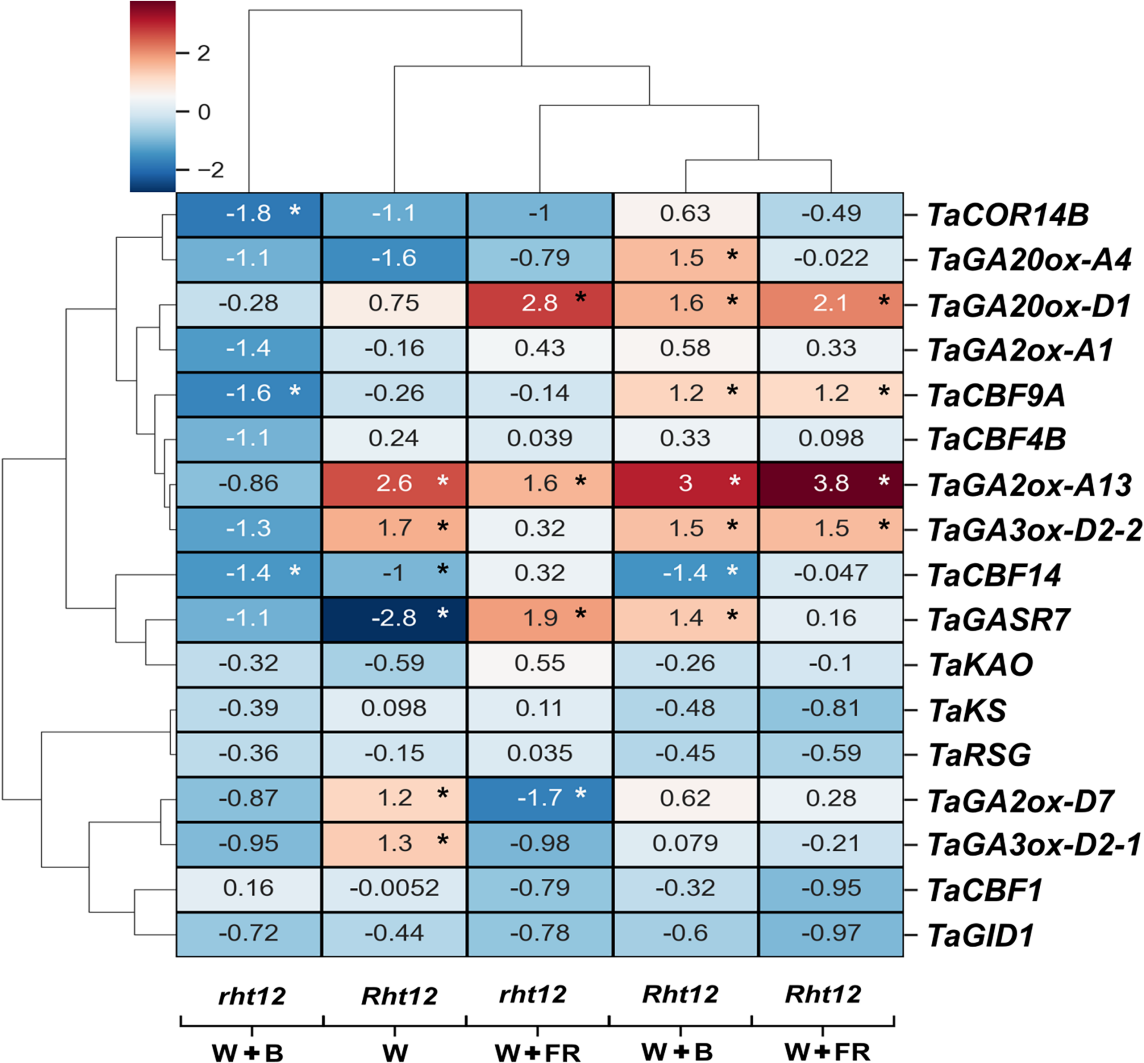
The effect of modified spectral conditions on the relative expression of the genes related to GA metabolism and cold acclimation in the tall (*rht12*) and dwarf (*Rht12*) wheat leaves after 10 days at 15°C. Transcript levels were calculated with the ΔΔCt method. Log_2_ expression values (Log_2_FC) are shown. W: white light (control), W+B: blue light enrichment, W+FR: far‐red light enrichment. *TaKS: ent*‐Kaurene Synthase, *TaKAO*: *ent*‐Kaurenoic Acid Oxidase, *TaRSG*: Repression of Shoot Growth gene, *TaGASR7*: Gibberellic Acid‐Stimulated Regulator 7. The data originated from three biological replicates. The values on the X and Y axes outside of the heat map refer to the distance or proximity of data after hierarchical clustering (Pearson). Values indicated by asterisks are significantly different from the control *rht12* (white light, W) at the level of *p*≤0.05. The detailed statistical analysis of the gene expression data can be found in Table S3.

All relative expression values were compared to the *rht12* line under W at 15°C. The results are shown on the heatmap (Figure 7) and with detailed statistical analysis in Table S3. During gene expression analysis, we focused on genes related to GA metabolism and cold acclimation. Most of the investigated genes were upregulated or remained unchanged under control conditions in *Rht12* leaves compared to *rht12*. Contrastingly, both *TaCBF14* and *TaGASR7* showed lower expression levels in the dwarfing line under W. Based on cluster analysis, W+B and W+FR treatments showed a closer relationship in the alteration of the expression patterns of the investigated genes in *Rht12* leaves. Interestingly, gene expression patterns in *rht12* leaves under W+B enrichment clustered into a distinct group with a greater distance from the light treatment effects on *Rht12*.

Among the genes involved in the initial part of GA biosynthesis, the *TaKS*, *TaKAO*, and *TaRSG* expression were unaffected by the treatments in both lines. In the case of late GA biosynthetic genes, *TaGA20ox‐A4* expression was higher after W+B treatment in *Rht12* compared to control illumination. *TaGA20ox‐D1* showed higher expression after W+FR treatment in both genotypes compared to the control *rht12*. This was also observed in the *Rht12* line exposed to W+B light. *TaGA3ox‐D2‐2* showed higher expression in the dwarfing line independently from light conditions compared to the wild type.

Among the investigated *GA2‐oxidases* (*TaGA2ox‐D7*, *TaGA2ox‐A13*, and *TaGA2ox‐A1*), which play a major role in GA deactivation, *TaGA2ox‐A13* expression levels were higher in *Rht12* compared to the wild type independently from the treatments. Interestingly, in *rht12* leaves, *TaGA2ox‐A13* upregulated after W+FR treatment. Besides, W+FR had a significant negative effect on *TaGA2ox‐D7* expression in *rht12*, while under W light, its expression was higher in *Rht12* compared to the control.

FR enrichment induced the putative GA‐inducible *TaGASR7* expression in *rht12*, whereas in *Rht12*, B light supplementation triggered this effect. Additionally, *TaGASR7* expression was lower in *Rht12* under W compared to the wild type, while its expression did not change after W+FR treatment in the dwarfing line.

The expression patterns of key regulators of cold acclimation, *TaCBF1*, *TaCBF4B*, *TaCBF9A*, and *TaCBF14*, were also investigated. Among these, the expression of *TaCBF1* and *TaCBF4B* did not change significantly after the treatments in the genotypes studied. However, the transcript level of *TaCBF9A* was significantly elevated by FR and B enrichment in the *Rht12* line, whereas its level was significantly reduced after B enrichment in *rht12* compared to the wild‐type control. The expression of *TaCBF14* was lowered in the dwarfing line under W and W+B treatments and also by W+B treatment in the wild type compared to the *rht12* control. Interestingly, W+FR exposure abolished this decrease in both lines. W+B treatment decreased *TaCOR14b* expression in *rht12*, whereas no other significant changes were observed in the genotypes tested.

### 3.8 Correlation network analysis of the presented data

**FIGURE 8:**
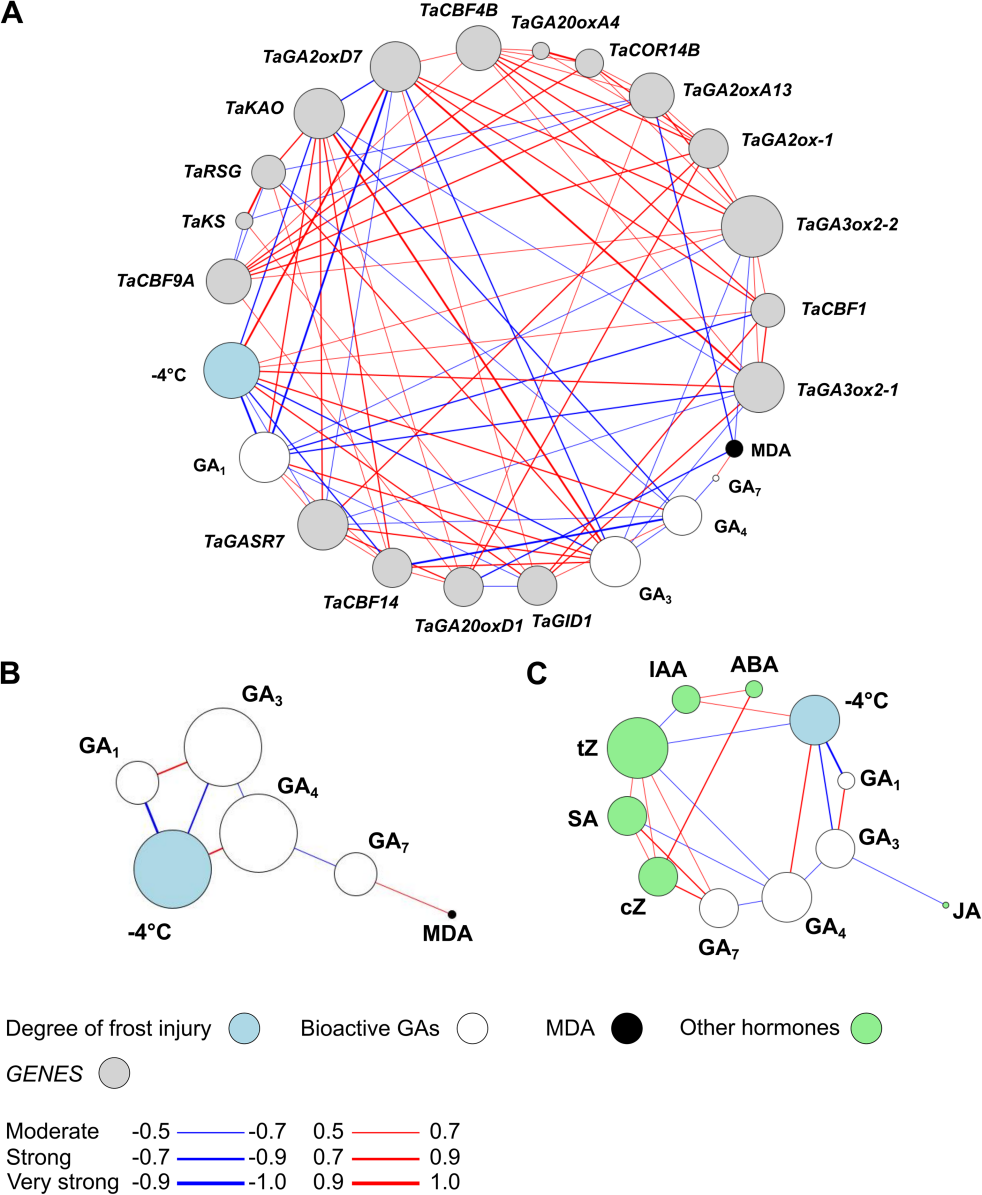
Correlation network diagrams illustrate relationships among investigated gene expression (grey), bioactive GAs (white), degree of frost injury (blue), and MDA (black) in (A); bioactive GAs, degree of frost injury and MDA in (B); and other hormones (green) with bioactive GAs in (C). Correlation analysis was performed using Pearson's correlation coefficient and the network was created with yED Graph Editor. Relationships were categorized into three ranges based on the correlation coefficient: Moderate (0.5–0.7), Strong (0.7–0.9), and Very Strong (0.9–1.0). Red lines indicate positive correlations, while blue lines indicate negative correlations. Line thickness represents the correlation strength, and circle size reflects the number of connections.

To show the connection between the presented datasets, we performed correlation network analysis (Figure 8). The degree of freezing injury at ‐4°C showed a strong negative correlation with GA_1_ and GA_3_ accumulation and *TaCBF14* transcript abundance, while a moderate negative correlation with *TaGASR7* (Figure 8A). Consequently, GA_1_ and GA_3_ showed a positive correlation with *TaGASR7* gene expression. GA_1_ and GA_3_ content also showed a negative correlation with *TaGA2oxD7*, which showed a positive correlation with frost injury at ‐4°C. MDA accumulation showed a strong negative correlation with *TaCBF14* gene expression and a very strong negative correlation with *TaGA2oxA13* transcript abundance, however, MDA did not show a correlative relationship with the degree of frost injury at ‐4°C. *TaGA2oxD7* gene expression pattern showed a very strong positive correlation with the degree of frost injury at ‐4°C. The strong correlative relationship between GA_1_ and GA_3_ and frost injury at ‐4°C is also shown in Figure 8B. While GA_3_ and GA_1_ correlated with JA accumulation, they formed a distinct group among the other hormones (Figure 8C). GA_7_ showed a correlative relationship with SA, tZ, and cZ. Analysis showed a positive correlation between IAA concentration and frost injury at ‐4°C.

## 4 DISCUSSION

In this study, the responses of winter wheat cultivar MH – tall, wild‐type *rht12* – and a dwarf, *Rht12* NIL, were compared after different light supplementation treatments (W+B or W+FR) for 10 days, at 15°C. Plant height, leaf frost tolerance (ion leakage, lipid peroxidation), hormonal pools as well as gibberellin metabolism and cold acclimation‐related gene expression were monitored. Neither of the light treatments was able to rescue the dwarf phenotype of the *Rht12*; however, FR supplementation enhanced the height of wild‐type plants compared to W. Such an effect on plant height is a well‐known phenomenon in response to neighbour proximity (increment in FR proportion due to reflection without the decrease of light intensity) during the SAS (Novák et al., 2016; Colombo et al., 2022). Interestingly, W+B also triggered an increment in plant height compared to control illumination, but it was less pronounced.

*Rht12* leaves were more sensitive to frost treatment at ‐4°C than the wild type under control illumination, which is similar to the findings of Szalai et al. (2022) in gibberellin‐insensitive *Rht* NILs. These observations support the hypothesis about the involvement of *Rht* alleles and GA in the freezing tolerance of wheat and also showed that GA‐related growth reduction does not increase the basal freezing tolerance of the leaves. The pre‐hardening‐inducing effect of FR enrichment in cereals at 15°C has long been known (Novák et al., 2016; Kovács et al., 2020; Ahres et al., 2021, 2023, 2025). In good agreement with this, W+FR treatment enhanced the freezing tolerance of the leaves of the *rht12* at both ‐4 and ‐6°C. However, the information about the relevant effect of W+B exposure on cereals is sparse. It was shown in winter barley (Ahres et al., 2023) or wheat leaves (Ahres et al., 2025) that frost injury was diminished when the W+FR light mixture was further enriched with a very narrow‐band B (λ_max_=410 nm) LED light source at 15 or 5°C, but this effect was highly dependent on genetic background in cereals. There is some evidence showing that monochromic B light could affect the freezing tolerance of Arabidopsis at low temperature (Imai et al., 2021; Li et al., 2021; Kameniarová et al., 2022). In the case of cereals, the relevant knowledge is limited (Crosatti et al., 1999; Novák et al., 2017; Ahres et al., 2025). Nevertheless, in the case of the *rht12* genotype, W+B treatment did not trigger the same effect as W+FR on the degree of frost injury. Interestingly, in *Rht12* leaves, the enrichment of W light with either B or FR was able to rescue the frost‐sensitive phenotype after freezing at the minimal temperature of ‐4 and ‐6°C was too severe for *Rht12* leaves, thus the beneficial effect of W+FR was not observed at this minimal temperature, unlike in wild‐type leaf segments. Consistently to the literature, W+FR enhanced the freezing tolerance of leaf tissues in both lines to withstand temperatures below 1‐2°C than their initial tolerance without cold exposure (Novák et al., 2016; Ahres et al., 2025). Similarly to W+FR, the beneficiary effect of W+B treatment on *Rht12* leaves was not detected at ‐6°C.

MDA accumulation was investigated before and after the freezing of leaf segments. Interestingly, at 15°C, MDA concentration was lower in the *Rht12* leaves than in the tall genotype under control conditions, which could be an undiscovered phenotype of *Rht12*. Since *Rht12* is reported to be a higher yielding, more lodging‐resistant genotype compared to *rht12* (Rebetzke et al., 2012), this observation might not be surprising. Some studies have demonstrated the possible role of RCS under physiological conditions in plant tissues, but this is not yet fully understood (Alché, 2019). However, W+FR illumination decreased the difference in MDA accumulation between the two genotypes at 15°C. After freezing at ‐4°C, the analysis showed a ~37.5% and a ~27% increase in W‐illuminated *rht12* and *Rht12* leaves, respectively, compared to their unfrozen level, thus *Rht12* showed lower MDA accumulation than the wild type. While MDA accumulation usually correlates well with tissue damage even under severe stress (Alché, 2019), at high levels of freezing injury or relative electrolyte leakage in the tissues, MDA levels might not correlate well with tissue damage or plant survival in cereals (Kolupaev et al., 2015; Sun et al., 2017), suggesting that MDA should be interpreted together with conductance measurements under severe freezing conditions. Similarly, MDA levels showed inconsistent changes with the frost injury of the leaf tissues under ‐6°C. However, W+FR illumination significantly alleviated MDA production in both genotypes, decreasing it close to their respective unfrozen levels. Ashgar et al. (2021) showed that growing young wheat under FR‐supplemented illumination reduced the GSSG level (thus also the GSSG/GSH ratio) in the frost‐sensitive genotypes. In our system, B supplementation did not affect MDA accumulation under freezing at ‐4°C in the wild type, while slightly increased it in the dwarfing line compared to its own MDA level at 15°C.

To explore the possible molecular changes behind the altered frost tolerance evoked by the treatments, we investigated the expression of cold acclimation‐related genes. In *Rht12* plants, we observed significantly lower *TaCBF14* expression under control conditions than in the wild type. It was found earlier that the *HvCBF14* mRNA transcript abundance determines cold acclimation capacity and winter hardiness in winter barley (Stockinger et al., 2007) and later in wheat during the W+FR induced pre‐hardening (Novák et al., 2016). In contrast to the significant increase in frost tolerance of the wild‐type leaves, none of the examined *CBFs* showed altered expression in response to W+FR treatment compared to the control conditions after 10 days. The *rht12* genotype in MH background possesses moderate frost tolerance (Prášilová & Prášil, 2001). It was also shown that frost‐sensitive genotypes of wheat accumulated lower levels of *TaCBF14* during cold acclimation than the tolerant ones (Asghar et al., 2021). It must be noted that *TaCBF14* expression showed time dependency in the winter wheat genotype Cheyenne during W+FR treatment at 15°C, with a peak of *TaCBF14* transcript accumulation during the earlier days of the treatment, in contrast to barley, where *HvCBF14* expression increased as time passed (Novák et al., 2016). Furthermore, other *CBFs* or *CBF*‐independent pathways might have participated in the W+FR‐induced pre‐hardening of *rht12* leaves. Interestingly, in the *Rht12* line W+FR treatment abolished the downregulation of *TaCBF14* compared to the W‐illuminated *Rht12* leaves. The degree of frost injury at ‐4°C also showed a strong negative correlation with *TaCBF14* gene expression. Simultaneously, W+FR triggered significantly higher *TaCBF9A* expression in *Rht12* compared to both genotypes under control illumination.

In winter wheat, einkorn, and barley genotypes, monochromatic B light‐induced the expression of *CBF14* more successfully after only 4‐h illumination compared to monochromatic red or FR light at 15°C (Novák et al., 2017). Similarly, B pulses induced higher *HvCOR14b* expression in barley (Crosatti et al., 1999). In contrast, both *TaCBF14* and *TaCOR14b* expression were downregulated by W+B exposure in *rht12* compared to control conditions. In the dwarf line, W+B exposure was unable to alter the lower *TaCBF14* expression, which was found to be an *Rht12* characteristic; however, the transcript accumulation of *TaCBF9A* became significantly higher compared to the wild type under W illumination.

Sun et al. (2019) found lower concentrations of bioactive GA precursors (GA_15_, GA_24_, GA_9_, GA_53_, GA_44_, GA_19_, GA_20_) in the dwarf *Rht12* NIL than in the wild‐type plants of a spring wheat variety. Our findings were similar only in the cases of GA_44_ and GA_9_ under control conditions. Interestingly, GA_19_ was significantly higher in the *Rht12* dwarfing line compared to the *rht12* leaves.

Most of the bioactive GAs, the GA precursors as well as the GA deactivation products were affected not only by the *Rht12* allele but also by the light treatments. Interestingly, the bioactive GAs were less influenced by B or FR enrichment in *Rht12* than in *rht12* leaves. W+FR increased GA biosynthesis in *rht12* leaves, which led to increased plant height (Pearce et al., 2016); however, this was not observed in *Rht12* lines due to insufficient bioactive GA content, despite the high amount of non‐bioactive precursor GA_19_ or GA_20_.

Both GA_1_ and GA_3_ accumulation was significantly higher in *rht12* leaves under W+FR and W+B light than under W. It must be highlighted that, due to the light treatments, GA_1_ concentration in *Rht12* leaves reached the level found in the wild type under control conditions. Higher GA_3_ content was also detected under W+FR in the *Rht12* NIL compared to W, but it was still far below the GA_3_ concentration in *rht12* leaves after W+FR treatment. This suggests a partial restoration of bioactive GA content in *Rht12* leaves by the light treatments, which might have contributed to the increased freezing tolerance of the dwarf line. Kosová et al. (2012) found that 7‐day‐long cold exposure to wheat increased leaf GA_1_ content in both spring and winter genotypes. Interestingly, GA_7_ content was unresponsive to light treatments in *Rht12* leaves, whereas GA_4_ levels were affected neither by the mutation nor by the light treatments. GA_4_ is suggested to be involved in vegetative growth (Sun et al., 2019) and might play a lesser role in light quality‐induced pre‐hardening. Although the potential early changes in bioactive GA concentration were not investigated in this study, these results are consistent with the findings of Kosová et al. (2012), who showed that bioactive gibberellins change dynamically during low temperature‐induced pre‐hardening of wheat instead of a linear decrease, suggesting a possible modulatory effect.

Sun et al. (2019) found that GA biosynthetic genes were generally upregulated in the *Rht12* plants compared to the wild type, which we also observed in the case of *TaGA3ox‐D2‐2* and *TaGA3ox‐D2‐1*, although the latter were more sensitive to the illumination spectrum. Under low R:FR ratio, wheat PHYB becomes inactive and the expression of several GA biosynthetic genes (e.g. *TaGA20ox1, TaGA20ox2* and *TaGA20ox4*) are released from repression (Pearce et al., 2016). In our system, *TaGA20ox‐D1* expression showed similar changes in both genotypes in the case of W+FR compared to W. Low R:FR ratio was reported to increase GA signalling in Arabidopsis and to decrease the abundance of DELLA proteins (Leone et al., 2014). Additionally, *TaGA20ox‐A4* showed higher expression in the dwarfing line in response to W+B treatment compared to both lines under W.

The expression of putative GA‐inducible *TaGASR7* was significantly lower in the *Rht12* leaves than in the wild type under control illumination. After W+FR treatment, *TaGASR7* expression was upregulated in *Rht12* leaves, reaching *rht12* control level. *TaGASR7* also showed higher expression in response to W+B treatment in *Rht12* leaves, compared to both untreated *Rht12* and wild‐type leaves. The investigated wheat *TaGASR7* gene was found to be homologous to petunia *PhGIP5*, which is gibberellin‐inducible and has a crucial role in cell division (Ben‐Nissan et al., 2004; Zhang et al., 2007). During the multiple‐sequence alignment of petunia GIP‐like proteins and their homologues from different plant species, *PhGIP5* was shown to be closely related to *AtGASA4* (Ben‐Nissan et al., 2004). *TaGASR7* is also listed as a possible orthologue of Arabidopsis *AtGASA4* based on sequence homology in the EnsemblPlants database. The overexpression of *AtGASA4* suppressed ROS accumulation in Arabidopsis (Rubinovich & Weiss, 2010). The tendency of *TaGASR7* expression was similar to the changes in MDA concentrations after ‐4°C freezing of W+FR leaf segments in both genotypes. The degree of frost injury at ‐4°C also correlated negatively with *TaGASR7* transcript abundance.

The *Rht12* mutation is reported to be able to affect GA deactivation pathways (Sun et al., 2019). However, low GA_8_ content could be a consequence of the similarly low GA_1_ content in the dwarfing line under control conditions. Interestingly, we also detected low GA_34_ concentration in the *Rht12* line under W light, whereas GA_4_ concentration was unaffected by the mutation. Both GA_1_→GA_8_ and GA_4_→GA_34_ metabolic changes are catalysed by the GA2ox enzyme family (Sun et al., 2019). The level of the deactivation metabolite GA_8_ was increased only by W+FR supplementation in both lines. At the same time, GA_34_ showed a great decrease under W+B and an even more drastic drop after W+FR treatments in *rht12* leaves. *TaGA2ox‐A13* expression was upregulated under W+FR supplementation in the *rht12* leaves and under all light conditions in the *Rht12* line. This gene was found to be upregulated in *Rht12* NILs and hypothesised to be responsible for the decreased bioactive GA content in these lines (Sun et al., 2019; Buss et al., 2020). Sun et al. (2019) found reduced *TaGA2ox‐A13* expression in response to exogenous GA_3_ treatment in *Rht12* NILs, however, it was still higher than in the wild‐type lines. In our system, W+B unaffected and W+FR further enhanced the expression of this gene in the dwarfing line compared to W treated *Rht12*, suggesting that the compensatory mechanism of W+B and W+FR treatments on GA accumulation was not related to the downregulation of this gene. The expression of *TaGA2ox‐D7* was higher in *Rht12* leaves under control illumination compared to the wild type. Additionally, it showed downregulation in wild‐type leaves treated with W+FR, compared to W treated *rht12* leaves. Interestingly, upon W+B and W+FR treatments, *TaGA2ox‐D7* transcript abundance was similar to W treated *rht12* leaves. *TaGA2oxD7* expression showed a negative correlation between GA_1_ and GA_3_ content during the experiment, and consequently, its transcript abundance had a positive correlation with frost injury.

In *Rht12* leaves, different hormone accumulation patterns were detected in the case of ABA, IAA, JA, SA, *t*Z or *c*Z, as most of their concentrations were lower compared to *rht12* leaves, except for the elevated levels of IAA and JA. Decreased ABA content may reflect the fact that it is generally not the absolute hormone levels but their ratios that are important. This relationship is quite frequent in the case of ABA and GA. Thus, diminished GA content in the *Rht12* genotype may be associated with lower ABA content, similarly as in the case of the CK/ABA ratio in cytokinin oxidase/dehydrogenase transformants, in which not only CK but also ABA content is lower (Macková et al., 2013).

Interestingly, the 10‐day‐long W+FR treatment decreased the level of ABA, SA, and *c*Z in *rht12* leaves. It has also been reported in einkorn wheat that the accumulation patterns of different hormones during the time course of cold acclimation could differ; thus, maximal hormone levels, for example in the case of ABA, might appear earlier (Vanková et al., 2014). However, Janeczko et al. (2018) found no difference between the ABA levels of cold‐acclimated, frost‐susceptible, or tolerant winter wheat genotypes. Contrastingly, the maintenance of significantly higher ABA levels in the leaves of other cold‐acclimated winter or spring wheat cultivars compared to non‐acclimated plants was also reported (Kosová et al., 2012). This phenomenon was also observed in winter barley prolongedly illuminated by light with a low R:FR ratio (Ahres et al., 2021, 2023). It has also been reported that an increased FR proportion of the incident light could induce cytokinin degradation in wheat (Lei et al., 2022). Decreased bioactive cytokinin accumulation reportedly also occurred during the cold acclimation of winter wheat (Kosová et al., 2012). The ABA decrease in the *Rht12* leaves was associated with an increase in JA, especially in the case of W+B. JA elevation may reflect a compensatory mechanism between ABA and JA observed in leaves of cold‐stressed rice (Jarošová et al., 2024). Those leaves exhibited low levels of ABA but high JA content, in contrast to roots, which had high ABA but low JA. Additionally, JA accumulation was found to be decreased by prolonged FR enrichment in winter barley (Ahres et al., 2021, 2023). JA could also contribute to frost tolerance through the ICE1‐COR‐CBF pathway; however, recent evidence advises the revalidation of the role of ICE1 in the process (Thomashow & Torii, 2020; Kidokoro et al., 2022; Wang et al., 2023b). Nevertheless, upon the cold acclimation of winter wheat, exogenous methyl‐jasmonate treatment increased antioxidant activity, proline content, and the expression of *TaCOR* genes: *TaWCS19* and *TaWCS120* (Repkina et al., 2021). The gene expression of wheat ICE RECRYSTALLIZATION INHIBITION (IRI) protein (*TaIRI*) was reported to be cold‐ and exogenous methyl‐jasmonate‐inducible (Jin et al., 2022).

In winter barley, light with a low R:FR ratio reduced SA levels (Ahres et al., 2023). In contrast to the wild type, W+FR treatment increased SA accumulation in the *Rht12* leaves compared to W‐illuminated *Rht12* leaves. Elevated SA levels were also observed during the cold acclimation of winter wheat (Janda et al., 2007; Kosová et al., 2012). Exogenous SA pre‐treatment increased the freezing tolerance of spinach (Min & Arora, 2022). Furthermore, both W+FR and W+B treatment decreased the IAA concentration in the leaves of the NIL compared to control conditions. IAA accumulation showed a positive correlation with frost injury at ‐4°C. 1‐day long cold treatment decreased the IAA accumulation in both winter and spring wheat, but after 3 days, it increased in both genotypes. Contrastingly, from the 7^th^ day, IAA content decreased in the winter genotype and increased in the spring genotype (Kosová et al., 2012). FR supplementation could disrupt IAA accumulation through the inactivation of PHYB in wheat, as reported by Pearce et al. (2016).

In summary, most of the parameters highlighted above was affected by GA availability and/or light quality. The correlative relationship was shown between the degree of frost injury of leaf segments at ‐4°C, GA_1_ and GA_3_ accumulation and *TaCBF14* or *TaGASR7* gene expression levels. In addition to our observations, the importance of light signalling in GA homeostasis was also supported by earlier studies, elucidating that PHYA, PHYB and CRY1 are also involved in the regulation of GA metabolism or signal transduction (Foo et al., 2006; Xu et al., 2021; Yan et al., 2021).

Since the GA_3_‐sensitive *Rht12* MH NIL used in this study might contain unidentified genomic regions from the *Rht12* source genotype (Karcagi 522M7K), the effect of this genetic noise on the measured parameters cannot be ruled out. Nevertheless, in their work, Szalai et al. (2022) used the facultative UK wheat variety ‘April Bearded’ (AB) and its two derivative GA‐insensitive NILs harbouring *Rht‐B1b* – source variety: ‘SD1’ breeding line – and *Rht‐B1c* – source variety: ‘Minister Dwarf’ – (Flintham et al., 1997) and showed the increased freezing susceptibility of these NILs after three weeks of cold acclimation (Szalai et al., 2022). These alleles are located on the short arm of chromosome 4B (Flintham et al., 1997), while the *Rht12* region is located on chromosome 5A (Sun et al., 2019; Buss et al., 2020). Including the results presented in this study, the freezing‐sensitive phenotype was detected in *Rht* NILs of different backgrounds, containing alleles from different sources that interfere with GA signalling in different ways (GA‐insensitivity vs. GA‐deficiency). Furthermore, due to the observed compensatory mechanism induced by W+FR and W+B treatments in GA accumulation, it is very likely that this phenotype is related to impaired GA signalling. However, the freezing tolerance of cold‐exposed or cold‐acclimated *Rht12* NILs should be described in the future.

## 5 CONCLUSIONS

Leaves of the dwarf, gibberellin deficient *Rht12* NIL showed reduced basal freezing tolerance compared to the tall MH background containing wild‐type *rht12* allele. Our results indicated that *Rht12* leaves differs from the wild type not only in their bioactive GA content, but in the accumulation patterns of other hormones as well. W+FR treatment proved to be more effective in enhancing frost hardiness in the wild‐type leaves than W+B, including the increment of bioactive GA_1_ and GA_3_ accumulation, *TaGASR7* expression or lower lipid peroxidation compared to control conditions. In the *Rht12* genotype, the response of hormone accumulation to altered light spectra was shown to be different from that in wild‐type leaves. Although special light circumstances could not affect the dwarf phenotype of *Rht12*, they were efficient in the partial rescue of the frost‐susceptible phenotype by partially restoring GA_1_ or GA_3_ accumulation, altering the expression of frost tolerance‐related transcription factors *TaCBF9A* or *TaGASR7*, and the level or the ratio of some other hormones, including the activation of JA accumulation. These results provide additional evidence for the frost susceptible phenotype of NILs harbouring dominant *Rht* alleles, suggesting that this effect is related to gibberellin. Our results also predict that the alteration of light signalling in wheat might compensate for the frost sensitivity of gibberellin‐deficient dwarf genotypes.

## AUTHOR CONTRIBUTIONS

Gábor Galiba, Péter Borbély, Zsolt Gulyás, Andreas Börner, and Radomíra Vanková wrote the manuscript; Gábor Galiba and Péter Borbély designed the research; Gábor Galiba, Péter Borbély and Danuše Tarkowská provided funding support; Zsolt Gulyás, Kitti Kulman, Zahra Tahmasebi, Mohamed Ahres and Tamás Pálmai performed the experiments; Radomíra Vanková, Petre Dobrev and Danuše Tarkowská performed the phytohormone measurements and evaluation; Gábor Galiba, Péter Borbély, and Zsolt Gulyás analysed the resulting data; Kristóf Jobbágy and Zsolt Gulyás made the figures.

## DECLARATION OF COMPETING INTEREST

The authors declare that they have no conflict of interest.

## Supporting information


**FIGURE S1.** Spectral composition of the applied light conditions.
**FIGURE S2**. MDA accumulation at ‐6°C minimal freezing temperature.

**TABLE S1.** Primers used in gene expression studies.

**TABLE S2.** Summary of the hormone analysis.

**TABLE S3.** Statistical analysis of gene expression data.

## Data Availability

All data supporting the findings of this study are available within the paper and in the supplemental data published online.
