# Supplementary material for: Blue or far‐red light supplementation induced pre‐hardening in the leaves of the Rht12 wheat dwarfing line: hormonal changes and freezing tolerance
Source: Physiol Plant. 2025 Mar 3;177(2):e70112. doi: 10.1111/ppl.70112 (PMC11876090; doi:10.1111/ppl.70112)
Supplement: Supplementary file 1 — FIGURE S1. Spectral composition of the applied light conditions. FIGURE S2. MDA accumulation at ‐6°C minimal freezing temperature. [file PPL-177-e70112-s002.pdf]

Blue or far-red light supplementation induced pre-hardening in the leaves of the *Rht12* wheat dwarfing line: hormonal changes and freezing tolerance

Zsolt Gulyás[1], Mohamed Ahres[1*], Tamás Pálmai[1], Kitti Kulman[1,2], Zahra Tahmasebi[1,3], Kalpita Singh[1,2], Kristóf Jobbágy[1,4], Danuše Tarkowská[5], Petre Dobrev[6], Radomíra Vanková[6], Péter Borbély[1*], Andreas Börner[7], Gábor Galiba[1,8]

[1]Agricultural Institute, Centre for Agricultural Research, HUN-REN, H-2462, Martonvásár, Hungary

[2]Doctoral School of Plant Sciences, MATE Hungarian University of Agricultural and Life Sciences, H-2100, Gödöllő, Hungary

[3]Festetics Doctoral School, MATE Hungarian University of Agricultural and Life Sciences, H-8360, Keszthely, Hungary

[4]Doctoral School of Biology and Institute of Biology, ELTE Eötvös Loránd University, H-1053, Budapest, Hungary

[5]Laboratory of Growth Regulators, Faculty of Science, Palacký University Olomouc and Institute of Experimental Botany, the Czech Academy of Sciences, CZ-78371 Olomouc, Czech Republic

[6]Laboratory of Hormonal Regulations in Plants, Institute of Experimental Botany of the Czech Academy of Sciences, CZ-165 02 Prague, Czech Republic

[7]Leibnitz Institute of Plant Genetics and Crop Plant Research, 06466 Seeland, OT Gatersleben, Germany

[8]Department of Agronomy, MATE Hungarian University of Agricultural and Life Sciences, GEORGIKON Campus, H-8360, Keszthely, Hungary

*Correspondence

Péter Borbély

E-mail: borbely.peter@atk.hun-ren.hu

Mohamed Ahres

E-mail: mohamed.ahres@atk.hun-ren.hu

Supplementary figures

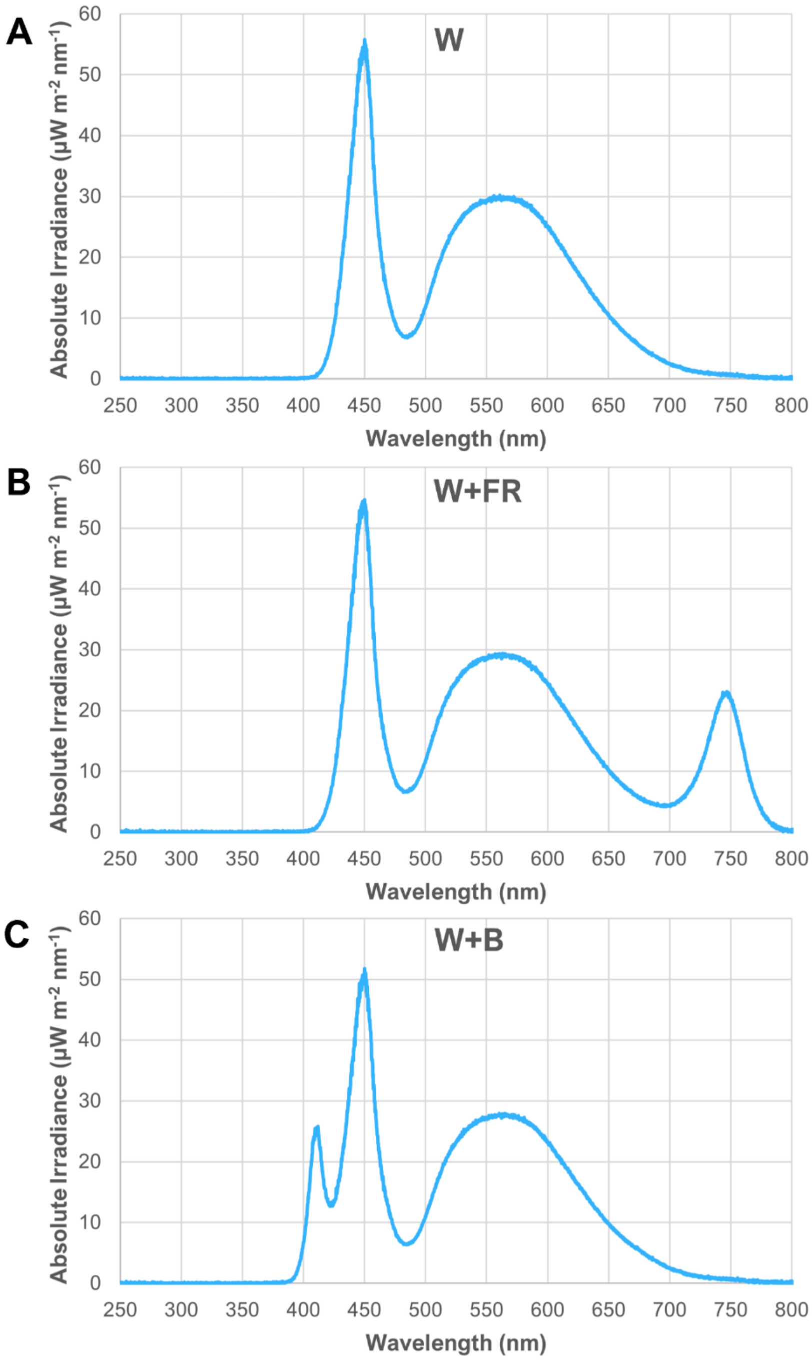

Figure S1. Spectral composition of light treatments at PPFD 250 µmol m^{-2} s^{-1}. A: White light (W); B: White light supplemented with Far-Red light (W+FR); C: White light enriched with monochromatic Blue (λ_{max}=410 nm) light (W+B).

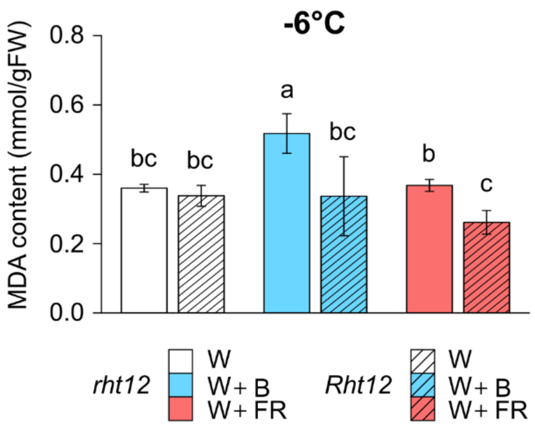

Figure S2. The effect of modified spectral conditions on lipid peroxidation at -6°C minimal temperature in the tall (*rht12*) and dwarf (*Rht12*) wheat leaf segments. The samples were collected to measure MDA content subsequently to the freezing tests implemented after 10 days of light supplementation treatments at 15°C. W: white light (control, white colour), W+B: blue light enrichment (blue colour), W+FR: far-red light enrichment (red colour). Solid bars: *rht12*; stripped bars: *Rht12*. Values marked with different letters are significantly different from each other at the level of $p \leq 0.05$ (n=3).
